# Genomics as a Clinical Decision Support Tool for Identifying and Addressing Modifiable Causes of Cognitive Decline and Improving Outcomes: Proof of Concept Support for This Personalized Medicine Strategy

**DOI:** 10.3389/fnagi.2022.862362

**Published:** 2022-04-18

**Authors:** Sharon Hausman-Cohen, Carol Bilich, Sandeep Kapoor, Eduardo Maristany, Anne Stefani, Alexandra Wilcox

**Affiliations:** ^1^IntellxxDNA, Austin, TX, United States; ^2^Kapoor Medical, Studio City, CA, United States; ^3^Naples Center for Functional Medicine, Naples, FL, United States; ^4^Resilient Health, Austin, TX, United States

**Keywords:** Alzheimer’s, genomics, clinical decision support, APOE ε4, precision medicine, personalized medicine, natural products, MCI

## Abstract

The landscape of therapeutics for mild cognitive impairment and dementia is quite limited. While many single-agent trials of pharmaceuticals have been conducted, these trials have repeatedly been unable to show improvement in cognition. It is hypothesized that because Alzheimer’s, like many other chronic illnesses, is not a monogenic illness, but is instead caused by the downstream effects of an individual’s genetic variants interacting with each other, the environment, and lifestyle, that improving outcomes will require a personalized, precision medicine approach. This approach requires identifying and then addressing contributing genomic and other factors specific to each individual in a simultaneous fashion. Until recently, the utility of genomics as part of clinical decision-making for Alzheimer’s and cognitive decline has been limited by the lack of availability of a genomic platform designed specifically to evaluate factors contributing to cognitive decline and how to respond to these factors The clinical decision support (CDS) platform used in the cases presented focuses on common variants that relate to topics including, but not limited to brain inflammation, amyloid processing, nutrient carriers, brain ischemia, oxidative stress, and detoxification pathways. Potential interventions based on the scientific literature were included in the CDS, but the final decision on what interventions to apply were chosen by each patient’s physician. Interventions included supplements with “generally regarded as safe (GRAS)” rating, along with targeted diet and lifestyle modifications. We hypothesize that a personalized genomically targeted approach can improve outcomes for individuals with mild cognitive impairment who are at high risk of Alzheimer’s. The cases presented in this report represent a subset of cases from three physicians’ offices and are meant to provide initial proof of concept data demonstrating the efficacy of this method and provide support for this hypothesis. These patients were at elevated risk for Alzheimer’s due to their apolipoprotein E ε4 status. While further prospective and controlled trials need to be done, initial case reports are encouraging and lend support to this hypothesis of the benefit of a genomically targeted personalized medicine approach to improve outcomes in individuals with cognitive decline who are at high risk for Alzheimer’s.

## Introduction

An estimated 6.2 million individuals are living with Alzheimer’s in the United States alone (Alzheimer’s Association, 2021). Additionally, mild cognitive impairment (MCI) prevalence has been estimated at approximately 5.9% of the adult population worldwide (Sachdev et al., 2015) and has been found in 3.7–13% of individuals aged 60–64, depending on the criteria used (Kumar et al., 2005). Individuals with MCI, progress to Alzheimer’s or other forms of dementia at a high rate. In some cases, the risk of progression is over 25% per year (Kumar et al., 2005; Petersen et al., 2018).

In 2020, approximately 305 billion dollars was spent on Alzheimer’s and dementia treatment in the United States alone (Wong, 2020). In addition to the direct cost of treating Alzheimer’s, there are the indirect costs including the economic burden put upon family members and caregivers and lost wages for younger individuals. Thus, even preventing the progression from MCI to Alzheimer’s in a portion of individuals, could represent huge cost savings to the United States and world health systems. Prevention of cognitive decline would also provide benefit for patients and their families, in both financial and emotional realms.

In the field of Alzheimer’s, the common “pill for an ill” strategy has proven to be highly unsuccessful. Over 200 Alzheimer’s drug trials have failed (Yiannopoulou et al., 2019). The current dogma in neurology is that Alzheimer’s disease (AD) and MCI most often provide a “one-way street” toward a decline in function. In fact, the current standard used to measure a therapeutic’s success in AD or MCI is a reduction of decline (Haeberlein et al., 2020). There is a need to switch the focus from slowing what is often perceived as an “inevitable decline” to the potential for improvement. Novel approaches to this global problem are needed, including looking at modifiable contributing factors.

It has been hypothesized that AD and MCI are not due to one cause, but instead multiple contributing factors and that to improve outcomes in Alzheimer’s and MCI these multiple contributing factors must be addressed simultaneously (Bredesen et al., 2018). In the manuscript presented here, we take the hypothesis one step further and suggest that the lack of success in identifying a cure, or even a treatment for Alzheimer’s, is because common diseases of aging such as cognitive decline, are due to a collection of genetic predispositions interacting with diet, environment, and lifestyle. Many of these predispositions can be identified using a well-curated genomics CDS. We present evidence that when root causes of cognitive decline are identified using genomics and addressed using genomically targeted interventions, improvements in cognition and the trajectory of the disease can be significantly changed.

An important point to consider in working with individuals with cognitive decline who have been labeled as “early Alzheimer’s” is the potential for this diagnosis to be incorrect. The ApoE ε4 genomic variant is found in over 60% of the patients diagnosed with Alzheimer’s (Riedel et al., 2016). However, even in the presence of ApoE ε4 and cognitive decline together are insufficient criteria for diagnosing Alzheimer’s. The INDIA-florbetapir study showed 79% of individuals, who had been presumptively diagnosed with Alzheimer’s, did not have evidence of Alzheimer’s when the gold standard amyloid positron emission tomography (PET) scan was done (Boccardi et al., 2016). Although all three of the individuals presented in this manuscript had ApoE ε4 variants (and one was ApoE ε4/4), in this vein, it would be wrong to assume that this means that their cognitive decline is definitively all due to “Alzheimer’s” and evidence of irreversible neurodegeneration. This brings up the question of what is causing these individuals’ cognitive decline, and could there be some contributing reversible factors? In this sampling of case studies, we aim to show how the IntellxxDNA*™* genomics Clinical Decision Support (CDS) platform can help clinicians identify potentially modifiable factors contributing to cognitive decline. Factors evaluated included genomics contributing to specific nutrient deficiencies, decreased ability to remove toxicants, genomics that contribute to proper amyloid processing, mitochondrial function, oxidative stress, and predispositions toward brain ischemia due to clotting cascade related variants or other pathways (Armstrong, 2019). As evidenced by the last case presented, genomics can also be used to optimize function and outcomes in cases of patients with cognitive decline who are amyloid PET scan positive. Even though these patients would be considered to have presumptive early Alzheimer’s, genomics can identify additional contributing factors, to help stabilize and improve cognition.

This proof-of-concept work, showing reversal of cognitive decline both in amyloid PET scan positive and other individuals is significant in that it shifts the paradigm from viewing Alzheimer’s and cognitive decline as a non-reversible condition to a treatable condition. It also presents a diverging hypothesis that genomically targeted personalized treatment plans may provide better outcomes than the standard of care options currently available.

## Materials and Methods

Patients were invited to participate through their clinician’s offices. All the clinicians were trained in integrative or functional medicine and were comfortable with the concept of complex chronic illness being due to a multitude of genomic variants that can have a combinatorial effect. The specific genomic CDS platform used by all three of the physician’s whose cases are reported here, was IntellxxDNA. The IntellxxDNA CDS is a scientifically rigorous genomics platform which consists of a highly cultivated collection of variants that have been shown to be clinically significant, in the form of reports categorized by specific health concerns. The CDS was developed by a team of physicians and researchers who conducted a broad review of rigorously published peer-reviewed human genomic scientific trials that looked at clinically significant genomic variants with good statistical significance related to Alzheimer’s and cognitive decline.

The methodology of IntellxxDNA has been described elsewhere, in previous publications (Way et al., 2021), but central to the reports is that only genomic variants that have been shown to be highly clinically significant in the published peer reviewed scientific literature as determined by *p* values [*p* < 0.05 for group with variant compared to control group in disease specific studies and <5 × 10^–8^ for Genome Wide Association Studies (GWAS)] were included. Thus, the composition of the CDS reflects current evidence for genetic findings that stand up to statistical rigor related to AD and other causes of dementia.

For further rigor in our health condition specific related reports, and to keep the report a manageable size, the variants chosen for these “odds ratio reports” needed to have had evidence for them increasing or decreasing the risk of Alzheimer’s or the particular disease state described by at least 20% or an odds ratio of at least 1.2. This decreased the list of candidate genes down from hundreds for each panel to just the dozen or so that are most significant. Many of the variants in the report have odds ratios of more than 2, representing a 100% increase in risk for individuals with that genetic variant. Combinations of variants that have been shown in the literature to have significant interactions are also reported. Another key factor for inclusion in the CDS is that variants displayed are determined to be potentially modifiable, not pathogenic.

All patients received the “Executive Combination Report” which is comprised of two smaller reports, the “Brain Optimization Report,” and the “Medical Overview Report.” The Brain Optimization report consists of over 15 different panels. The “capstone” panels in the Brain Optimization report focus on gene variants highly associated with AD, leukoaraiosis, and brain ischemia. Thus, the composition of the CDS reflects current evidence for genetic findings that stand up to statistical rigor related to AD and other causes of cognitive decline. The platform also includes variants related to hormonal factors, nutritional factors, inflammatory pathways, as well as genetic variants related to gluten intolerance, intestinal barrier function, and removal of toxicants. The Medical Overview report focuses on underlying contributing factors to other chronic medical conditions such as cardiac disease and obesity.

In an effort to learn more about the mechanisms of action of the genomic pathways and their associations with cognitive decline, the physicians underwent genomic education which was provided to them as part of the CDS support platform both in the form of live and online learning modules. They were also educated regarding the following information: combinations of gene-gene variant interactions or interactions between the genetic variants, how an individual’s genomics could play into various environmental exposures including exposures to toxins and toxicants. Physicians were also educated regarding the mechanisms of supplements, vitamins, and other interventions as well as their epigenetic effects on the expression of various gene products or regulation of gene products. The clinical studies supporting the evidence for the potential interventions presented in the CDS were also covered in the supporting educational platform. Finally, the epigenetic potential of an individual’s diet, nutrient intake and other lifestyle choices was recognized.

As a CDS, the purpose of the platform is to present, to physicians, information found in the published medical literature relating to these gene variants along with information regarding the numerous options for potentially responding to these variants (Potential interventions). Every sentence of the genomics CDS is thusly based on the published literature and is referenced, again conveying a high level of scientific rigor to the platform. The CDS thus allows the physician to gain better understanding of potential contributing root causes to the patient’s cognitive decline and how these various root causes might be addressed.

Patient DNA was obtained via salivary collection device and then analyzed using a combination of an Affymetrix custom array and polymerase chain reaction (PCR) at RUCDR, a clinical laboratory amendments (CLIA)/College of American Pathologist (CAP) certified lab. The data was then transmitted to IntellxxDNA*™* and each patient’s genomic information was then presented to the physician via the IntellxxDNA*™* CDS platform. The report format consists of the explanation of the single nucleotide polymorphism (SNP) function, how it relates to various clinically relevant conditions, along with referenced potential intervention and modification strategies.

In addition to genomic sequencing, appropriate labs were obtained by each of the patient’s treating physicians, which were ordered at the physician’s discretion and specific to each patient’s history, physical exam, and chief complaints. These labs were addressed by each physician in their usual manner of practice.

### Discussion of the Development of Potential Interventions

As a CDS platform, the genomics platform does not give patient specific treatment recommendations. Rather, it supports the clinician in better understanding the published medical literature so s/he can make better, more targeted choices with respect to the potential interventions.

Potential intervention mechanisms for variants can take many forms. Examples include the following: (1) Supplying cofactors to pathways that are not working efficiently. An example is the glutathione peroxidase 1 variant (GPX1), where the GPX1 cofactor is selenium. Selenium is found in large amounts in Brazil nuts. Thus, adding 3–7 Brazil nuts to the diet each week or supplementing with selenium would be an included potential intervention for this variant (Stoffaneller and Morse, 2015; Cardoso et al., 2016). (2) Addressing a genomic weakness. For example, if a patient has an Interleukin 6 (IL6) SNP that has been associated with increased levels of IL6, utilizing a supplement such as curcumin that has been shown to lower IL6 and cross the blood-brain barrier would be a way to help overcome this genomic “weaknesses” (Ghandadi and Sahebkar, 2017). Another example is in raising substrate levels, such as increasing B12 levels to overcome deficiencies in transcobalamin 2 (TCN2), which is the main carrier of B12 to the central nervous system (Mitchell et al., 2014). Potential interventions listed in the cognition and memory pathway necessarily target the particular genetic variant being addressed and also have to show evidence in the literature for improving cognitive outcomes. This same genomics CDS and same method of identifying and developing a treatment plan targeted to genomic variants has been shown to be effective in individuals with autism spectrum disorder and other neurodevelopmental concerns (Way et al., 2021). While the capstone panels in the IntellxxDNA neurodevelopment report are, of course different, there is considerable overlap in the panels supporting nutrition, detoxification, oxidative stress, and inflammation.

For assessing cognitive function, three different assessment tools were utilized based on the preferences of the clinician.

1) Saint Louis University Memory Screener (SLUMS): A well-recognized and standardized screening test that efficiently and quickly identifies mild cognitive impairment as well as dementia. On the SLUMS, normal cognition is represented by a score of 27–30. Mild cognitive impairment is relayed by a score of 21–26 and dementia is correlated with a score of 20 or less (Adogwa et al., 2018).
2) The Montreal Cognitive Assessment Screener (MoCA). The MoCA is another cognitive screening tool to screen for mild cognitive impairment and provides a benefit for testing individuals with MCI over the SLUMS screening test, in that it has a wide number of versions that prevent “memorizing” the test (Lebedeva et al., 2016). For the MoCA, a score of 26 and above is considered normal, and less than 20 is consistent with dementia (Horton et al., 2015).
3) CNS vitals were also used in some of the case studies. The CNS Vital Signs tool neurocognition test is broken down into about 8–15 subsections, depending on which indices are measured, with scoring displayed graphically as well as numerically with colors of dark green (above average), green (average), yellow (low average), orange (low), and red (very low) (Littleton et al., 2015).

## Case Studies to Support This Hypothesis

Results from three case studies are presented below. The first case is presented with detail to illustrate how the platform is used and the subsequent cases are presented with more brevity, highlighting the most important findings.

### Case 1: Mr. A

#### History and Chief Complaint

Mr. A is a 79-year-old male. He is a retired phone operator with a chief complaint of short-term memory loss and “brain fog” which he has noticed for approximately 10 months. Other complaints include low energy, fatigue, and poor sleep as well as nocturia. He presents with his partner of 35 years, and they are both worried that he is experiencing early symptoms of Alzheimer’s Disease. He is a longtime patient of the physician’s practice.

In particular, he has noted difficulty following conversations and is forgetting names and words. He is now feeling embarrassed in social situations. Prior to this he was social with his partner and enjoyed hosting and cooking special dinners with friends. The pandemic did decrease dinners with friends, but he also began having trouble cooking because he forgot how to make dishes that used to be his staples. He also stopped cooking for himself and his partner because he felt he could not remember the key ingredients. In lieu of cooking, they started ordering in with high frequency.

#### Social History

• Education: 2-year associates degree
• Smoking status: Non-smoker
• Alcohol and substance use: Occasional wine with dinner

#### Family History

He has a family history of Coronary Artery disease, Peripheral Artery Disease, and Alzheimer’s disease.

#### Past Medical History

He has a past medical history of hyperlipidemia, benign prostatic hypertrophy, and mildly overweight with a body mass index (BMI) of 26.4. Body type characteristic of central obesity.

#### Initial Cognitive Evaluation

His initial cognitive evaluation showed him to have mild cognitive impairment with initial SLUMS score of 23/30 and MoCA score of 23/30. On both scales, a score of less than 20 is considered consistent with dementia. Baseline screening for depression was also done utilizing a Patient Health Questionnaire (PHQ9) depression screener and the score was 2 consistent with no symptoms of depression (Kroenke et al., 2001).

#### Initial Laboratory Results

The initial serum laboratory results are shown in Table 1. Previous limited genetic testing indicated that he is heterozygous for ApoE ε4 and the well-known methylenetetrahydrofolate reductase variant, (MTHFR) C677T. Most of the labs were unremarkable except for significantly low testosterone and elevated lipoprotein a [Lp(a)]. Low-density lipoprotein (LDL) was minimally elevated, but LDL particle number was high, consistent with small particles which are more atherogenic. High sensitivity c-reactive protein (hs-CRP) and homocysteine were on the high side of ideal although still considered normal. There was no evidence of heavy metals in serum or urine.

**TABLE 1:** Initial serum laboratory values for Mr. A.

Normal	Mildly elevated	Significantly elevated	Low levels
HDL: 59 mg/dL	LDL: 125 g/dL	Lp(a): 239 mg/dL	Testosterone: 290 ng/dL
Vitamin D: 53.2 ng/mL	hs-CRP: 1.7 mg/L	LDL-P: 1,984 nmol/L	Omega Index: 6.7%
Lp-PLA2: 143 nmol/min/mL	Homocysteine 8.9 μmol/L		Omega 6/3 ratio: 5.5
Hemoglobin A1C: 4.9%	Copper/Zinc Ratio: 1.4		
TSH: 1.73 μIU/mL			
Magnesium: 2.2 mg/dL			
No heavy metals detected			

*HDL, high-density lipoprotein; g/dL, milligrams per deciliter; ng/mL, nanograms per milliliter; Lp-PLA2, lipoprotein-associated phospholipase-A2; nmol/min/mL, nanomoles per minute per milliliter; TSH, thyroid stimulating hormone; μIU/mL, micro-international units per milliliter; LDL, low-density lipoprotein; hs-CRP, high-sensitivity C-reactive protein; mg/L, milligrams per liter; μmol/L, micromoles per liter; Lp(a), lipoprotein(a); LDL-P, low-density lipoprotein particle number; nmol/L, nanomoles per liter; ng/dL, nanograms per deciliter.*

#### Initial Medication and Supplements

Initial medications and supplements were limited to vitamin C, multivitamin, B12/folate, vitamin D with K2, magnesium, and zinc every other day.

#### Advanced Genomics Obtained

Genomics were obtained through the IntellxxDNA Clinical Decision Support platform, as described above. The Brain Optimization and Medical Overview reports were ordered and showed a wide variety of modifiable genomic factors that can contribute to cognitive decline and overall wellness. The most salient variants are discussed in detail below.

#### Discussion of Genomic Results

Genomic analysis was done. The specific variants which the patient had were presented in an organized fashion displaying their relationship to health issues such as cognition and memory, cardiac disease, brain ischemia, blood sugar, etc. Priority was given to addressing the variants based on their relevance to the chief complaints of the patient and the prevalence of the gene variant, with less prevalent gene variants given higher emphasis. The platform gives information on over 600 genomic variants. The variants present that were determined to be most significant to this patient are discussed below but for purposes of brevity and practicality, not all variants will be discussed.

##### ApoE ε4

This patient had a known “ApoE ε4,” which is often thought of as an “Alzheimer’s gene” variant. At least one allele of ApoE ε4 is present in over 60% of people with Alzheimer’s (Riedel et al., 2016). This variant was addressed utilizing a “ketoflex” diet. A ketoflex diet is a high fat, mildly ketogenic diet that focuses primarily on highly nutritious whole foods, with many of the fat sources being plant-based. Lifestyle changes of proper sleep and exercise (swimming), brain exercises (Brain HQ), and stress reduction methods were prescribed. The importance of these interventions in ApoE ε4 individuals with early Alzheimer’s or cognitive decline has been discussed and published previously by Dr. Dale Bredesen (Bredesen et al., 2016; Bredesen et al., 2018; Toups et al., 2021).

Additionally, because ApoE ε4 codes for a lipoprotein that interacts with 1,700 different gene promoters, there are many other interventions that make sense for individuals with ApoE ε4 that were referenced by the CDS, and some of these were selected and prescribed (Liao et al., 2017). ApoE ε4 conveys less ability to properly clear and cleave amyloid (Kim et al., 2009). Ashwagandha, which supports the clearance of amyloid was chosen as an intervention for this patient. Ashwagandha works in part by conveying neuroprotective effects against beta amyloid induced neuro-pathogenesis (Kurapati et al., 2013). Ashwagandha has been correlated with increased neurite outgrowth and decreased neuronal degradation and neurite retraction, as well as less programmed cell death (Kuboyama et al., 2005; Kuboyama et al., 2020). Ashwagandha has also been shown to upregulate the LDL receptor which enhances amyloid-beta clearance in animal models and has been shown to decrease neurodegeneration (Sehgal et al., 2012). It has been shown to improve memory in human subjects (Choudhary et al., 2017). It also has other mechanisms that can be particularly beneficial for ApoE ε4 individuals in that ashwagandha helps to decrease the buildup of amyloid-beta by decreasing the β-secretase (BACE) activity (Kuboyama et al., 2014). In addition to human studies supporting improvements in cognition, ashwagandha ameliorated cognitive deficits in aged transgenic mice with amyloid precursor protein (APP)/presenilin 1 (PSEN1) variants known to be associated with dementia and other animal models (Kuboyama et al., 2005; Sehgal et al., 2012).

Individuals with ApoE ε4 have also been shown to have higher levels of tumor necrosis factor-alpha (TNFα), IL-6, and interleukin 1 beta (IL-1β) (Fan et al., 2017). The patient’s magnesium was switched to magnesium threonate from magnesium glycinate, as magnesium threonate has been shown to better cross the blood-brain barrier (BBB), decrease TNFα expression, and to improve cognitive outcomes in individuals with dementia (Wang et al., 2013; Wroolie et al., 2017; Yu et al., 2018).

##### Peroxisome Proliferative Activated Receptor Gamma Coactivator 1 Alpha

Peroxisome proliferative activated receptor gamma coactivator 1 alpha (PPARGC1A) is associated with mitochondrial biogenesis (Lehman et al., 2000). ApoE ε4 individuals have compromised mitochondrial function (Yin et al., 2019). Given that the patient was an ApoE ε4 and had two variants in a PPARGC1A SNP (Lehman et al., 2000; Cheema et al., 2015), a mitochondrial support vitamin that contained extra alpha-lipoic acid, acetyl L-carnitine, *N*-acetylcysteine, and other mitochondrial cofactors was substituted for his usual multivitamin (Wang et al., 2010; Wright et al., 2015).

##### MTHFR/IL6 Combination

The patient’s genomics revealed numerous vascular dementia risk factors, including two copies of an IL6 variant. This variant has been shown to increase the risk of vascular dementia by 3.7x when combined with one or more copies of MTHFR C677T, which the patient also had (Mansoori et al., 2012). The patient was already on a B vitamin with methylfolate, but IL6 had not been addressed by the initial regimen. To address this IL6 variant, omega 3s and a bioactive form of curcumin were prescribed. Both omega 3s and curcumin have been shown to lower IL6 (Blaylock and Maroon, 2012; Ramirez-Ramirez et al., 2013) and to improve measurements of cognitive decline (Sarker and Franks, 2018; Martí Del Moral and Fortique, 2019). An added benefit of curcumin is that it can help promote proper processing of APP so less amyloid beta builds up in the brain (Blaylock and Maroon, 2012). Other IL6 lowering interventions were also added such as Epigallocatechin gallate (EGCG) and dietary capsaicin (Mueller et al., 2010; Pervin et al., 2018).

##### Protein Kinase C Eta

Other genes were elucidated by the genomic CDS that this clinician identified to be potentially contributing factors that would not normally have been on his radar. The CDS was able to highlight genomic factors related to brain ischemia. One such factor was identified to be a variant in Protein Kinase C Eta (PRKCH), which contributes to brain ischemic risk. This PRKCH variant is only found in less than 3% of the population and has been associated with a 37% increase in stroke risk (Sherry et al., 1999; Wu et al., 2009). Protein kinase C eta is involved in tight junction integrity and variants in this gene relate to hemorrhagic and lacunar stroke risks (Willis et al., 2010; Haffner et al., 2016). Citicoline, which has been shown to improve tight junctions and decrease stroke risk, was prescribed (Fioravanti and Buckley, 2006; Martynov and Gusev, 2015). Citicoline has multiple studies showing improvement in cognition endpoints for individuals with vascular dementia risk (Gareri et al., 2015). Saffron (*Crocus Sativus*) was added to patient’s regimen as another known potential intervention that both crosses and tightens the BBB (Batarseh et al., 2017). Saffron has been shown to improve cognition in human studies compared to placebo. In fact, it has outcomes data equal to donepezil or memantine in human trials of individuals with MCI/AD (Avgerinos et al., 2020). Saffron has been shown to reduce amyloid beta load in animal models (Batarseh et al., 2017).

##### Platelet Aggregation and Tissue Plasminogen Activator Related Variants

Other hidden genomic contributing factors that were revealed by the CDS include Glycoprotein 3 a (GP3a) and Lipoprotein A (LPA). GP3a stimulates platelet aggregation and the variant the patient had relates to more sticky platelets (Kucharska-Newton et al., 2011). The LPA gene variant which the patient had is known to lead to elevated Lp(a) levels and decrease the amount of natural tissue plasminogen activator in the blood (Ueda et al., 2018). While the LPA gene variant is most known for its ability to increase cardiac risk approximately 2-fold (Clarke et al., 2009), it has also been shown to increase stroke risk to a lesser extent (Langsted et al., 2019). Based on these cardiovascular genomic risk factors, he was started on baby aspirin which has been shown to directly lower Lp(a) levels (Ranga et al., 2007). Low dose atorvastatin was prescribed to address overall cardiovascular and vascular dementia risk factors (Geifman et al., 2017).

##### Glutathione and Detox Related Variants

The removal of toxins and toxicants via glutathione transferases is important for maintaining brain health and cognition. When there are variants or deletions in these pathways, heavy metals, and other toxicants such as pesticides and herbicides can build up in the brain, compromising brain heath (de Mendonça et al., 2016; Wang et al., 2016; Andreoli and Sprovieri, 2017). The patient was found to have Glutathione S-Transferase mu 1 (GSTM1) null (i.e., both copies of this gene are absent), Glutathione S-Transferase theta 1 (GSTT1) null, as well as two copies of the Glutathione S-Transferase pi 1 (GSTP1) Ile105Val variant, which is highly clinically significant. Both the GSTM1 null and GSTP1 homozygous Ile105Val have been associated with a significantly higher risk of AD (Wang et al., 2016). The cognitive effects of GSTM1 null are amplified when combined with ApoE ε4 conveying 3.07x the risk of dementia as well as 2.1x the risk of atherosclerosis (de Mendonça et al., 2016; Grubisa et al., 2018). GSTP1 Ile105Val has been shown to be associated with a 1.87x the risk of AD/MCI and greater susceptibility to the negative effects from lead exposure (Wang et al., 2016). Thus, supporting this individual’s glutathione pathways became paramount. *N*-acetylcysteine (NAC) was chosen as an intervention for this patient as it has been shown to directly increase glutathione levels (Moradi et al., 2009).

#### Seven Months Follow Up for Mr. A

Seven months after the patient’s protocol was changed to address his specific genomic findings, as identified by the CDS, the patient showed marked improvement. Given the specificity of his genomics, and the ability for his clinician to discuss these findings, the patient and his partner were motivated to incorporate recommended diet, lifestyle, and supplement regimens into their routine. He did extremely well, rapidly noticing improvement in his cognitive abilities. The physician retested the patient with MoCA (using different version) and SLUMS at this time and the patient showed notable improvement, now scoring a 26 on MoCA (previously 23) and 27 on SLUMS (previously 23). Additionally, significant improvements were seen in laboratory parameters as shown in Table 2.

**TABLE 2:** Comparison of pre- vs. post-intervention serum laboratory values for Mr. A.

Lab	Pre-intervention	Post-intervention
Lp-PLA2	143 nmol/min/mL	109 nmol/min/mL**
hs-CRP	1.7 mg/L	1.2 mg/L**
Total cholesterol	204 mg/dL	180 mg/dL*
LDL	125 mg/dL	104 mg/dL*
LDL-P	1,984 nmol/L	1,325 nmol/L**
SMLDL-P	605 nmol/L	375 nmol/L**
HDL	59 mg/dL	60 mg/dL*
Lp(a)	239 μmol/L	196 μmol/L*
Homocysteine	8.9 μmol/L	9.3 μmol/L
Hemoglobin A1C	4.9%	5.0%
Fasting glucose	82 mg/dL	90 mg/dL
Vitamin D	53.2 ng/mL	68.7 ng/mL**
Omega index	6.7%	8.9% **
Omega 6/3 ratio	5.5	4.5*
Testosterone	290 ng/dL	440 ng/dL**
Magnesium	2.2 mg.dL	2.2 mg/dL
Copper	100 mcg/dL	98
Zinc	72 mcg/dL	88 mcg/dL*
Copper/Zinc ratio	1.4	1.1
TSH	1.73 μIU/mL	1.07 μIU/mL**
Free T4	1.4 ng/dL	1.6 ng/dL*
Free T3	3.0 pg/mL	3.5 pg/mL*

*Denotes more than 10% improvement. **Denotes more than 20% improvement. Lp-PLA2, lipoprotein-associated phospholipase-A2; hs-CRP, hs-CRP, high-sensitivity C-reactive protein; LDL, low-density lipoprotein; LDL-P, low-density lipoprotein particle number; SMLDL-P, small low-density lipoprotein particle number; HDL, high-density lipoprotein; Lp(a), lipoprotein(a), TSH, thyroid stimulating hormone; Free T4, free thyroxine; Free T3, free triiodothyronine; nmol/min/mL, nanomoles per minute per milliliter; mg/L, milligrams per liter; mg/dL, milligrams per deciliter; nmol/L, nanomoles per liter; μmol/L, micromoles per liter; ng/mL, nanograms per milliliter; ng/dL, nanograms per deciliter; mcg/dL, micrograms per deciliter; μIU/mL, micro-international units per milliliter; pg/mL, picograms per milliliter.

During follow up visits, the patient noticed that he feels more energetic, empowered, and motivated to achieve further improvements in his memory and wellbeing. He is reassured and sees that he can improve his memory, not just prevent further decline. Recognizing the improvement was also reassuring and motivating to his partner.

**Specific improvements noted by patient and his partner**

• His mood is very improved. He feels his “glass is half full” rather than half empty-part of this he attributes to pandemic getting better, but he also feels that the improvements in his diet, exercise and supplement regimen are worthwhile for him.
• His energy is improved, and he is sleeping better.
• He has returned to previous hobbies and interests as well as cooking. He has become especially creative with his Keto-flex diet. He feels like he can remember the key ingredients for the dishes and even created a new keto-flex friendly chili-con-carne recipe. Once again, they are entertaining friends, and he is enjoying cooking for these parties. The patient has even begun teaching his friends how to eat well so they can also lose their extra abdominal fat and improve their weight. His partner notes that the patient is less fearful of social interactions, is remembering more words and names and is less embarrassed by his condition.

### Case 2: Mr. B

#### History and Chief Complaint

Mr. B, at presentation in December of 2020, was a 70-year-old man status/post mantle cell lymphoma in 2017 treated successfully with chemotherapy and an autologous stem cell transplant. He presents complaining of worsening “brain fog” ever since chemotherapy 3 years ago. Extensive intake was done where questions were asked regarding ability to function with activities of daily living, motivation, energy, mood, and memory symptoms as well as screenings for a wide variety of physical and mental health concerns. There was no evidence of depression or underlying mood disorder so full PHQ9 was not done. Baseline labs were essentially within normal limits other than sex hormone labs which are discussed below. Thus, other labs are not reported in detail in this case.

A detailed initial cognitive assessment was done using the CNS vital signs platform, which showed his neurocognitive index to be in the 1st percentile. This patient, upon presentation, had nine areas in the red or very low category, 1 yellow (low), 2 orange (low average), 2 green (average), and 2 dark green (above average).

#### Social History

• Occupation: Firefighter chief, on the 911 response team; also has a history of working in the military on nuclear submarines.
• Smoking Status: Currently non-smoker, rarely smoked in early years but high lifetime smoke exposure as he was a firefighter.
• Alcohol and substance use: None
• The patient is one of 13 children

#### Family History

He had a family history of a mother with hypertension. She died of dementia at 89. His father had myocardial infarction/heart disease and colon cancer. His father died at 70. Siblings: Two of his 12 siblings had thyroid cancer, one brother with heart disease, severe atopic dermatitis, and arthritis.

#### Past Medical History and Initial Treatment

His past medical history was significant for Mantle Cell Lymphoma as above. From a cognitive standpoint, a magnetic resonance imaging (MRI) from June of 2021 showed no acute findings but did report mild to moderate chronic small ischemic changes and white matter disease. Volumetric interpretation using the NeuroQuant platform showed whole brain volume to be in the 32nd percentile with mild to moderate global atrophy, but hippocampal volume was in the 57.00% on left and 44.00% on the right, giving an asymmetry index of 0.26 (70.00%). The report from the radiologist concluded, “These findings are *not* strongly suggestive of neurodegenerative/Alzheimer’s type dementia.”

Based upon the patient’s presenting symptoms and medical history, the treating physician believed that he was likely to have vascular factors contributing to his cognitive decline as well as strong components of neuronal damage and toxicity from the various chemotherapies and high lifetime environmental exposures due to his work as fire chief and on nuclear submarines. He had received combination chemotherapy which is BiCNU (Carmustine), Etoposide, Ara-C (Cytarabine), and Melphalan. The mechanisms by which chemotherapy may contribute to cognitive decline are many. Chemotherapy can contribute to oxidative stress, vascular damage, and decreased cerebrovascular perfusion. White matter abnormalities such as demyelination and activation of microglia, alterations in neuronal signaling, as well as direct neuronal injuries from chemotherapy can contribute to neurodegeneration and immune dysregulation (Mounier et al., 2020). Thus, hyperbaric oxygen therapy (HBOT) was recommended as a first-line treatment to address some of these perfusion issues and other issues (Shapira et al., 2021). The patient consented to the risks and benefits of HBOT and started on 10 sessions of HBOT (Heyboer et al., 2017).

In 2/21, 1 month after presentation and ten sessions of HBOT, the patient’s CNS vitals were repeated. The patient’s Neurocognitive index increased from the first to the third percentile. However, category wise this correlated to a significant difference with only having four in the low “red zone,” instead of the nine indices originally measuring in this low red range. Additionally, the patient subjectively reported noticeable improvement in the efficiency of running errands and activities of daily living. Due to seeing improvement with HBOT, the patient wanted to know if there were more specific interventions that could be recommended. Thus, “Brain Optimization” genomics were ordered to help give insight into other potential contributing factors to his cognitive decline that could be optimized.

#### Discussion of Genomic Results

In 5/21 the patient returned for review of genomics. The genomics findings were very informative. Many of the patient’s underlying genomic “weaknesses” overlapped with mechanisms that could have been exacerbated by the neuronal effects of chemotherapy and compounding the patient’s symptoms of cognitive decline.

##### ApoE ε4

The patient was found to be an ApoE ε3/4. As described in the published literature, ApoE ε4 individuals do not use sugar well as a brain energy source (Krikorian et al., 2012; Wu et al., 2018). They also have evidence of increased mitochondrial oxidative stress, which can lead to mitochondrial damage, and would benefit from lifestyle changes that support mitochondria (Simonovitch et al., 2019). In this case, a diet very low in sugar, as well as intermittent fasting was recommended (Zhang et al., 2017; Morrill and Gibas, 2019).

##### Glutathione Transferase

Like Mr. A, in the case above, Mr. B was also found to have significant issues in his glutathione transferase-related pathways with deletions of both GSTT1 and GSTM1 as well as homozygous variants in GSTP1. *N*-acetylcysteine (NAC) was started (1,200 mg a day), as well as glutathione using a combination of daily oral and weekly IV glutathione treatments to promote detoxification (Moradi et al., 2009; Hauser et al., 2009; Pizzorno, 2014; de Mendonça et al., 2016).

##### Endothelial Nitric Oxide Synthetase and Matrix Metalloprotease

Homozygous variants in two highly significant genomic pathways were noted: Endothelial nitric oxide synthetase (NOS3) and matrix metalloprotease (MMP13). These two pathways are known to strongly contribute to white matter changes in the brain, known as leukoaraiosis. Homozygosity for this NOS3 (endothelial NOS) variant has been shown to increase the risk of leukoaraiosis by 290%. Additionally, he was homozygous for an MMP13 variant which increased the risk of leukoaraiosis by 390% (Fernandez-Cadenas et al., 2011). Leukoaraiosis has been shown to have a significant demyelinating component and to contribute to cognitive decline (Brown et al., 2007). One study found that the presence of leukoaraiosis correlated with a lower Mini-Mental-Status Exam (MMSE) score (Schmidt et al., 2007).

Endothelial nitric oxide synthetase is hypothesized to contribute to leukoaraiosis by increasing vascular constriction and decreasing blood flow (Hassan et al., 2004). Dietary changes to incorporate more beets, leafy greens, and other high nitric oxide producing foods were recommended (Lidder and Webb, 2013). HBOT is also a direct intervention for this pathway. One animal study found that 24 h after a 60-min session of HBOT, endothelial NOS transcribed protein product was increased by 60%, and endothelial NOS messenger RNA (mRNA) content by 20–30% (Xu et al., 2009). Twenty additional sessions of HBOT were done to address this pathway at 1.8 standard atmosphere (ATM).

Matrix metalloprotease is involved in tissue remodeling and blood-brain barrier permeability (Lakhan et al., 2013). Excessive MMP13 levels lead to the degradation of important components of the extracellular matrix, resulting in blood-brain barrier leakage (Lakhan et al., 2013; Yabluchanskiy et al., 2013). Two copies of the minor allele, carried by this patient are associated with increased MMP13 expression (Shi et al., 2016). With the purpose of supporting remyelination, Nicotinamide adenine dinucleotide (NAD), and citicoline were added to the regimen. NAD has been shown to potentially improve remyelination, improve brain adenosine triphosphate (ATP) production, and improve phospholipid metabolic pathways (Wang et al., 2017; Cuenoud et al., 2020). Citicoline was added to support remyelination and leukoaraiosis pathways, but also due to variants relating to decreased ability to synthesize choline (Martynov and Gusev, 2015; Feng et al., 2017).

##### Brain Derived Neurotrophic Factor

This patient had a known and significant variant in the brain derived neurotropic factor (BDNF) gene, found only in 11% of the population (Sherry et al., 1999). BDNF is an important nerve growth factor, and the patient’s variant has been associated with a 1.88x risk of cognitive impairment or Alzheimer’s (Sherry et al., 1999; Ji et al., 2015). One of the best-studied interventions for increasing mature BDNF, the synaptogenic promoting form, is intense exercise (de Assis and de Almondes, 2017). Thus, the patient was encouraged and agreed to add exercise to his regimen 3x/week. He was to maintain his heart rate at least at 120 beats per minute for 30 min.

##### Other Genomic Variants and Interventions

Other variants regarding adequate conversion of folate to the methylfolate, and TNFα mediated were also addressed in a targeted fashion with methylated B vitamins and *Hericium Erinaceus*, derived from lion’s mane mushroom (Li et al., 2018; Cajavilca et al., 2019).

Mr. B’s sex hormone levels were checked. Both testosterone and estradiol levels were found to be low. His initial total testosterone was 249, free testosterone was 30, and estradiol was less than 15. The grouping of hormonal receptors variants carried by Mr. B have been shown to interact adversely with ApoE ε4. Even though this study was done in women, estrogen still has hormonal and brain modulation effects in men. Since estrogens are derived directly from testosterone via aromatization, men require adequate testosterone to help maintain estrogen levels in the brain-protective range (Zimmerman et al., 2011; Fernández-Martínez et al., 2013). There is evidence that men with higher estrogen levels perform better on verbal memory tests compared to men who have lower estrogen levels (Zimmerman et al., 2011). Testosterone replacement was given which corrected both the testosterone and estradiol levels and post treatment levels were brought to total testosterone levels of 400, free testosterone levels of 65, and estrogen levels 20.

#### Summary of Regimen

• NAC 1,200 mg/day
• Glutathione 500 mg orally a day or 3 g IV weekly
• Multivitamin, Methylated B complex
• Citicoline 250 mg/day
• Testosterone replacement
• Lion’s Mane
• Checked environmental toxins/heavy metals
• 20 more HBOT sessions at 1.8 ATM × 60 min
• Exercise 3x a week, heart rate at least 120 beats per minute for at least 30 min

#### Three and Six Months Post-Genomic Follow-Ups for Mr. B

Three months after genomic interventions, significant improvements were noted. In 8/2021, the patient’s SLUMS Score was 24, up from a previous score of 20. This transitioned the patient from having dementia into the middle of the mild cognitive impairment range. He reported feeling better overall, was able to operate more functionally, and found it easier to do errands. He also noted less forgetfulness.

Six months after interventions were introduced, the patient was contacted by phone and stated that he felt that his memory was about 70% better. In addition, he reported that his writing ability has returned to normal. The patient was able to complete his taxes with little effort, and his energy levels had improved. He no longer noted any difficulties with daily activities, and in fact, had resumed higher energy activities that he previously enjoyed such as hunting with his son. He also reported that the chronic back pain that he had for years from a firefighting injury has also improved.

### Case 3: Ms. C

#### History and Chief Complaint

Ms. C is a 62-year-old female who presents to the office complaining of memory problems over a few years and had particularly noticed some worsening starting approximately 18 months prior to seeking help from her current physician. Her main complaint is that she has poor short-term memory; forgetting names; trouble remembering places gone the day before, as well as misplacing items that she used daily. The patient is extremely detail-oriented and quite bright. She graduated college in 3 years due to having so many credits and had a bachelor’s in science. The patient had initially joined a brain research study that was being offered through a local psychiatrist’s office. As part of the study, testing revealed two copies of ApoE ε4. PET brain scan indicated presence of amyloid. Amyloid PET scans have been shown to have a sensitivity of approximately 91% for AD (Marcus et al., 2014). She was aware that an amyloid positive PET scan is highly suggestive of developing Alzheimer’s and upon learning that, she decided to seek more progressive care with an integrative practice. In the same study, an MRI of the brain revealed a small amount of white matter changes, which discussed above, are an independent risk factor for a decreased score on cognitive testing (Schmidt et al., 2007).

This third case is an interesting case in that patient is at extraordinarily high risk for Alzheimer’s due to her being a known ApoE ε4/4. While there is some variation in risk based on other gene variants present or absent, individuals who are homozygous for ApoE ε4 have been shown to have as high as 14x the risk for Alzheimer’s (Farrer et al., 1997). Additionally, at the time of presentation (4/16/19), although the patient was only 62 years old and had a college education, she had a SLUMS of 21, which put her in the category of mild cognitive impairment bordering on mild dementia (20 and below is defined as dementia) (Adogwa et al., 2018). The patient was concerned about her future, given her strong family history of neurodegenerative diseases of both Alzheimer’s and Parkinson’s. Her personal history of essential tremor and restless legs provided additional worry. Essential tremor has been associated with some mild defects in executive function, memory, and cognition (Bermejo-Pareja, 2011). Restless legs have been linked to cerebral microvascular disease and gliosis. Additionally, restless legs are commonly associated with Parkinson’s (Bhalsing et al., 2013; Walters et al., 2021). Patient had past medical history of depression which was under control with treatment at the time of presentation to the clinic and during the course of treatment with PHQ2 and PHQ9 scores consistent with no evidence of depression or depression under full control.

#### Social History

• Education: She graduated college in 3 years due to having so many credits and had a bachelor’s degree in science.
• Smoking status: Non-smoker
• Alcohol and substance use: No history of substance use, drinks about 1 glass of wine 5 days per week.
• Moderate exerciser.

#### Family History

Mother: AD (onset age 61). Parkinson’s disease (onset 65). Deceased from AD age 77. Her mother also had migraines. Father with glioblastoma and hyperlipidemia. Maternal aunt with AD in her 70s and uterine cancer. Her two siblings are healthy.

#### Past Medical History

She has a past medical history of hyperlipidemia, subclinical hypothyroidism, essential tremor, restless legs, osteopenia, situational anxiety, depression, and eczema. Depression was under full control with PHQ 2 score of 0 when evaluated in 4/2019.

#### Baseline Medications and Supplements

Escitalopram 10 mg, Horizant extended release 600 mg, trazodone 50–100 mg, atorvastatin 10 mg, and Deplin (L-methylfolate) 15 mg, and NAC 600 mg.

#### Pertinent Initial Laboratory Results

Baseline labs were significant for elevated LDL, undetectable estradiol and testosterone, borderline low vitamin D, and elevated cortisol. See Table 3 below for comparison of initial and post treatment labs.

**TABLE 3:** Comparison of pre- vs. post-intervention serum laboratory values for Ms. C.

Lab	Pre-intervention	Post-intervention
LDL	140 mg/dL	96 mg/dL*
AM cortisol	22.9 mcg/dL	21.4 mcg/dL
Vitamin D	34 ng/mL	50 ng/mL*
Zinc	105 mcg/dL	79 mcg/dL
Copper	1,050 mcg/L	882 mcg/L
TSH	2.2 μIU/mL	0.966 μIU/mL*
Free T3	2.8 pg/mL	2.8 pg/mL
Hemoglobin A1C	5.5%	5.2% *
Vitamin B12	840 pg/mL	>2,000 pg/mL
Estradiol	<17.0 pg/mL	Ranged from 38 to 98.7 pg/mL *
FSH	141.8 IU/L	Ranged from 74 to 81 IU/L*
Testosterone	<12 ng/dL	Ranged from 110 to 179 ng/dL*
Heavy metals screen	Negative	

*Denotes significant change in levels that may relate to clinical improvement. LDL, low-density lipoprotein; TSH, thyroid stimulating hormone; Free T3, free triiodothyronine; FSH, follicle-stimulating hormone; mg/dL, milligrams per deciliter; mcg/dL, micrograms per deciliter; ng/mL, nanograms per milliliter; mcg/L, micrograms per liter; μIU/mL, micro-international units per milliliter; pg/mL, picograms per milliliter; IU/L, international units per liter; ng/dL, nanograms per deciliter.

#### Initial Interventions Prior to Genomics

Ms. C’s physician was trained in the “Reversal of Cognitive Decline” (ReCODE) protocol developed by Dr. Dale Bredesen. Thus prior to obtaining results of genomics, the treating physician started her on lifestyle interventions that have been shown to be beneficial to individuals with ApoE ε4. These included a mildly ketogenic diet, exercising with intervals of intensity, and fasting 13–16 h a day (de Assis and de Almondes, 2017; Zhang et al., 2017; Morrill and Gibas, 2019). As discussed in cases above, carrying the ApoE ε4 allele has been shown to be associated with improper cleavage of APP, mitochondrial dysfunction, oxidative stress, and TNFα mediated inflammation (Kim et al., 2009; Ramirez-Ramirez et al., 2013; Fan et al., 2017; Simonovitch et al., 2019). Thus, many of the basic interventions of the ReCODE protocol were also added empirically (Bredesen et al., 2016; Bredesen et al., 2018). These included a micronized bioavailable curcumin, Omega 3s, CDP-Choline, vitamin D3-K2, sulforaphane, mitochondrial support vitamin, coenzyme q10 (CoQ10), and magnesium threonate. At the time of presentation, the patient was taking methylfolate and NAC, as started by a previous physician. She was started on Ashwagandha to lower am cortisol and for brain benefit (Chandrasekhar et al., 2012).

#### Discussion of Initial Genomic Results

Six months later, on 10/08/19, the patient’s SLUMS had improved to 24. At this point the clinician had received the patient’s genomic results through the IntellxxDNA platform and accordingly adjusted her regimen.

##### Cyclooxygenase 1

Pyrroloquinoline Quinone (PQQ) was added to better enhance mitochondrial function and biogenesis (Chowanadisai et al., 2010) given the presence of a mitochondrial cyclooxygenase 1 (COX1) variant that has been shown to have an interactive additive effect with ApoE ε4 (Coto et al., 2011). This COX1 variant has been shown to convey reduction of cytochrome c oxidase complex IV activity. Reduced cytochrome c oxidase (complex IV) activity has been observed in post-mortem studies of AD, and particularly effects the temporal cortex and hippocampus (Wang and Brinton, 2016). While these mitochondrial variants overall increase AD by 52%, in ApoE ε4 individuals the risk of AD was shown to be increased by an astronomical 430%. This variant appears to create mitochondrial DNA replication instability (Coto et al., 2011). Mitochondrial methylation pathways were also of concern and supplement regimen was revised to include a combination of methylfolate and folinic acid.

#### Further Exploration of Genomics 2 Months Later

Two months later the patient was seen in a follow up. At this point, her husband was starting to notice improvement in the patient’s brain function and memory in terms of her ability to organize and complete tasks successfully and she was not asking questions and repeating questions as often. Genomics was explored further at this visit. The key topic discussed was the patient’s large number of glutathione-related pathways and detox pathways affected as well as her being a Human Leukocyte Antigen (HLA)-DQ2.5/2.2.

##### Human Leukocyte Antigen-DQ2.5/2.2

The patient also had HLA-DQ2.5/2.2. This combination put her at very high risk for gluten intolerance, with approximately 10 times the risk compared to the general population, a completely gluten-free diet was stressed (Almeida et al., 2016).

##### Other Pathways Addressed

For purposes of brevity, each of her detoxification-related pathways will not be discussed in detail, but detox pathways were addressed by adding intranasal glutathione in addition to optimizing her NAC and sulforaphane dosing (Moradi et al., 2009; Pizzorno, 2014; Yoshida et al., 2015).

Some of the other variants she had have been already addressed by initial supplements started. A few other genomically targeted optimizations were made, such as increasing magnesium and increasing the dose of omega 3s and switching to one that had eicosapentaenoic acid (EPA)/docosahexaenoic acid (DHA) and docosapentaenoic acid (DPA). Hormone-related pathways were addressed with BioTe estradiol and testosterone pellets, oral micronized natural progesterone, diindolylmethane (DIM), and iodine. Thyroid hormone replacement was given for a TSH of 4.99.

#### Fifteen, 22, and 28-Month Post Genomic Follow-Ups for Ms. C

At 15 months, the patient returned, and SLUMS had improved to 30 (perfect score). The patient at that time was, however, losing too much weight on the ketogenic diet and thus asked if she could switch to a Mediterranean diet. She also found the intranasal glutathione irritating and difficult to travel with and stopped this.

There was then a gap in patients returning to the clinic due to Covid and travel. When she returned to the clinic at 22 months, patient and husband self-reported some mild increase in memory concerns compared to the previous visit. The clinician switched to using MoCA due to having more version options and the score was 24. She was restarted on intranasal glutathione and protocol was slightly modified. Diet was kept as Mediterranean. It is unknown what triggered her decline as there were a lot of variables including covid vaccination, extensive travel with dietary changes, etc. as well as the potential for additional neurodegeneration due to her underlying pathology and biochemistry as an ApoE ε4/4 with positive amyloid PET scan.

Twenty-eight months after starting original protocol, her MoCA was still 24 (MoCA requires quite a long delay on the memory section of the test, meaning significant delay from the time patient is asked to remember five words to the time they are asked to recall these five words), but the patient’s SLUMS was back at 27 and CNS vitals showed her to be doing quite well with overall neurocognitive index of 30% and nine of the 12 indices showing her performing in the green – average category. Her verbal memory score is still significantly impaired in the red, very low range. The CNS vitals results fit with her own observations. The challenge for this patient remains with her verbal learning and verbal memory, which is often the case for individuals with ApoE ε4/4 or early Alzheimer’s (Bussè et al., 2017). The patient and her husband are pleased with her progress, compared to when she presented. They realize that maintaining her previous gains as an ApoE ε4/4 will require dedication to her regimen. She has restarted her mildly ketotic diet and is working closely with a nutritionist so she can maintain her weight while on this diet. She has also begun brain plasticity training games to improve her verbal memory.

## Discussion of Case Studies

It is well recognized that individuals carrying the ApoE ε4 allele are at high risk for cognitive decline and AD. The three case studies above, all purposely from individuals with ApoE ε4, illustrate that it is possible to improve indices of cognitive decline and improve patient well-being and function. These case studies also support the hypothesis that the typical downward trajectory of cognitive decline can be stabilized and reversed. The third case in this series is particularly encouraging, as significant gains were achieved even though the patient is an ApoE ε4/4, had a positive amyloid PET scan and had been accepted into a study, due to meeting the criteria for early Alzheimer’s.

To our knowledge, improvements in cognitive measures, especially with the magnitude of improvement seen in these patients who were optimized utilizing genomically targeted interventions, have not been previously reported in studies with any currently available pharmaceutical intervention for cognitive decline. In fact, the most recent drug approved drug for MCI or mild AD, Aducanumab, was approved based on showing no improvement in cognitive outcome measures, just less decline (Haeberlein et al., 2020).

We believe that the genomically targeted interventions provided superior outcomes for these patients because cognitive decline is due to a multitude of variables, rather than just one. This multi-pronged approach allows for many of these patient specific genomic variables to be addressed simultaneously. Alzheimer’s researcher and neurologist, Dr. Dale Bredesen, pointed out in his original discussion on the topic of why Alzheimer’s drugs have failed, that there are over 36 different contributing factors. This list has recently been further expanded. Identifying and treating as many of them as possible is necessary to achieve the desired outcome of improved cognition (Gustafson, 2015). These contributing factors are frequently compared to “holes in a roof.” If you were to patch only one or two holes in a roof with 36 holes, the rain would still enter, and you would not see a noticeable improvement. Over 215 different genes have been implicated in Alzheimer’s disease alone (Jansen et al., 2019). The advantage of a CDS for genomics is that it allows clinicians to identify and prioritize treatment recommendations to provide the best impact by identifying some of the potentially clinically significant genomic factors that are present in an individual. It is also important to note that the inclusion of diet and lifestyle changes discussed in the genomics CDS are an essential part of a “brain recovery protocol” as they are strong epigenetic modulators, as well as modulators of inflammatory and hormonal signaling (Sharma et al., 2020).

By addressing a multitude of variables identified by genomics including vascular risk factors, inflammation, brain permeability, various nutrient deficiencies, hormonal imbalances, difficulties with removing heavy metals, chemicals, and other neurotoxic substances, the above patients were able to gain significant improvements in their cognition. Each of these contributing factors has their independent effects on the brain and cognition, but they also are important to address due to their combinatorial effects. For example, elevated levels of brain inflammatory cytokines have been highly linked with cognitive decline due to the detrimental effects of inflammation on neuronal health, but also increase amyloid deposition (Wang et al., 2015). Mitochondrial deficiencies, such as those conveyed by the COX1 variant and intranuclear hormone receptors estrogen receptor (ESR) 1 and ESR2 and a multitude of other variants can interact directly with ApoE ε4, conveying an increased risk to individuals with these variants (Coto et al., 2011; Fernández-Martínez et al., 2013). While the individuals above shared the most well-established AD genomic variant (ApoE ε4), their regimens were customized, based on their other genomics, along with their lab values, personal, and family history.

That these case reports come from three independent physician offices helps to show the reproducibility of the benefits using a genomics CDS for improvement in cognitive decline. It also provides optimism and support for further larger, controlled trials. Limitations to the data presented here include the fact that no aggregate data is given as to how many patients each clinician has treated utilizing this approach and what the average magnitude of change in cognition scores would be for a larger population of individuals. With regards to this topic of what percentage of patients might benefit from this approach, we have presented some aggregate data from one of the practices (AS) in Table 4.

**TABLE 4:** Changes in cognition scores for series of 23 patients from one office.

Patient and direction of change	Pre-score	Post-score	Follow up months
1 – I	26	28	9
2 – I	19	26	8.5
3 – D	18	7	17
4 – I	21	28	6.5
5 – D	20	14	10
6 – N	20	19	11.5
7 – D	17	13	8
8 – D	17	13	13
9 – D	12	6	22
10 – D	14	9	23
11 – I	22	24	9
12 – I	13	16	6
13 – I	21	29	32.5
14 – I	21	27	33
15 – D	9	6	21.5
16 – N	25	25	19.5
17 – N	21	20	14
18 – N	26	26	24
19 – I	26	29	17.5
20 – I	20	22	15.5
21 – I	20	22	9.5
22 – N	16	15	7
23 – N	19	19	13

*I, improved cognitive score and function; D, decreased cognitive score and function. N, neutral (no significant change) in cognitive score or function.*

While not done with the intention of being a prospective trial, the office of Dr. AS did collect serial data on 24 patients from their practice. SLUMS test was used in the majority of the patients for monitoring of cognition, with MoCA used as the follow-up test in three cases. Each of these tests are measured on a 30-point scale. One patient was excluded from this analysis as during the course of treatment, she was diagnosed with frontotemporal dementia.

Of the 23 patients, 43.5% improved, with an average increase in their score of 4.2 points with an average duration of follow-up being 14.7 months. 26.1% of patients’ cognitive scores remained stable (between –1- and +1-point change on the 30 point scale) with a mean delta of –0.5 over 14.83 months. Finally, 30.4% of patients did not appear to benefit from this approach. In this group cognition decreased over time, consistent with what is seen classically in the Alzheimer’s and MCI literature, with an average drop of 5.57 points over 16.36 months.

Further studies with prospective analysis of variables such as the presence or absence of specific genomic variants, comorbid conditions, and patient compliance that may contribute to outcomes are needed. However, the raw data from the series of 23 patients presented, lends credence to the fact that the three cases presented are representative of an approach that can provide a reasonably high frequency of success. The ability to improve outcomes in 30–40% of a patient population with cognitive decline and to stabilize an additional 25% of cognitive decline patients would represent a very significant improvement to the available therapeutic milieu. One question the readers might ask, as they review the case reports, is whether there could be a placebo effect causing some of the gains since there was no control group. However, it is a reasonable conclusion, as these individuals were all ApoE ε4 positive, to use the published studies regarding mild cognitive impairment/early Alzheimer’s as “typical controls.” The natural course of AD as an illness or even MCI is continued worsening, particularly in ApoE ε4 individuals. Even using the treatment group for the Aducanumab study (as compared to their placebo group), no gains in cognitive measures were noted over an 18-month period, just 20% less loss (Haeberlein et al., 2020). Thus, the fact that these individuals showed significant improvement rather than decline, makes this method certainly worth exploring with further studies.

Another limitation of this approach is that patients in this study had the resources to afford supplements and high-quality nutritious foods as prescribed. If this approach were to be attempted in a more traditional medical practice with individuals of varying socio-economic classes this could be a barrier as supplements and interventions such as hyperbaric oxygen are not covered on Medicare, Medicaid, or commercial insurance plans. Given the tremendous cost of Alzheimer’s and dementia care absorbed by both the health care system, Medicare/Medicaid and an individual and his/her family, this barrier might eventually be addressed, in part, by detailed treatment cost analysis showing the potential tremendous cost savings of this approach in the long run which would provide good evidence for Medicare and other insurance companies to consider coverage of these interventions. In the meanwhile, since changes to health policy would take time, based on cost benefit ratios many families might find it not only worthwhile but cost effective to self pay for these interventions.

While the reproducibility of this method was shown amongst clinicians trained in integrative or functional medicine in this manuscript, a third limitation to this approach is that most clinicians are not comfortable with recommending supplements or natural products, even if they are over the counter and have a GRAS (generally regarded as safe) rating. This is due to lack of knowledge of the mechanisms, efficacy and supporting research and typical dosing for vitamins and supplements. This educational barrier can be overcome by appropriate educational platforms such as those used to educate the physicians above. Time constraints and other biases held by the physicians would need to be addressed through our continuing medical educational system before this approach could be utilized on a wide-spread scale.

Despite the barriers and potential problems with this genomically targeted approach, the potential demonstrated here for the reversal or improvement of cognitive decline is significant and should be explored further as it has tremendous societal and individual economic and emotional implications.

This approach is also ideal for addressing patients who want to focus on the prevention of cognitive decline for individuals with very mild symptoms of memory impairment that might otherwise be documented as “normal aging.” The fact that that many of the interventions for addressing these root causes are nutrients, supplements, and lifestyle modifications with excellent safety profiles, makes this approach well positioned to be used for prevention. A genomically targeted approach to cognitive decline is reproducible, efficacious, and also cost-effective. Further, because the patient realizes that the genomic data being discussed is uniquely their own, they have significantly greater compliance with recommendations made.

## Conclusion

A wide variety of hypotheses regarding Alzheimer’s have been presented in the recent literature. These include hypotheses and data supporting the involvement of inflammation, small vessel disease, and hypoperfusion (Hakim, 2021), the hypothesis regarding oxidative stress and mitochondrial dysfunction (Simonovitch et al., 2019), the hypothesis regarding the potential detrimental effects of environmental exposure to toxicants (Wang et al., 2016), the role of poor amyloid processing (Armstrong, 2019), and many more. All of these hypotheses have support and are likely true. However, not all of the identified contributing factors apply to each patient. To get optimal outcomes, the treating clinician needs to be able to identify which of the plethora of AD risk factors that have been elucidated in the literature are applicable in their particular patient. Genomics is a powerful tool that can help with this step. The second half of the equation, once identified, is what can be done about each of the potential contributing factors. Taking the contributing factor back to the genomic level helps in identifying modification strategies. A curated genomic CDS that identifies the underlying genomics contributing factors provides a framework for physicians to develop a personalized treatment plan. Genomics as a part of a CDS platform (in this case, IntellxxDNA) has been shown to be beneficial for improving outcomes in other complex neurological diseases such as autism (Way et al., 2021). There is now good evidence for the hypothesis that this same genomically targeted approach can be utilized for AD and MCI.

## Data Availability Statement

The original contributions presented in the study are included in the article/supplementary material, further inquiries can be directed to the corresponding author.

## Ethics Statement

Ethical review and approval was not required for the study on human participants in accordance with the local legislation and institutional requirements. The patients/participants provided their written informed consent to participate in this study. Written informed consent was obtained from the individual(s) for the publication of any potentially identifiable images or data included in this article.

## Author Contributions

SH-C and CB contributed to the conceptualization of this work. SH-C, SK, EM, and AS contributed to the methodology. SK, EM, and AS carried out the study investigation, as well as developed and supervised treatment plans, and collected data. SH-C, CB, and AW wrote the original draft and edited the manuscript. All authors have read and agreed to the published version of the manuscript.

## Conflict of Interest

AW is an employee at IntellxxDNA^TM^. IntellxxDNA^TM^ was the genomics clinical decision support tool used in this study but has no financial interests. SH-C is the medical director of IntellxxDNA and does have ownership interest. CB is the CEO of IntellxxDNA^TM^ and does have ownership interest. The remaining authors declare that the research was conducted in the absence of any commercial or financial relationships that could be construed as a potential conflict of interest.

## Publisher’s Note

All claims expressed in this article are solely those of the authors and do not necessarily represent those of their affiliated organizations, or those of the publisher, the editors and the reviewers. Any product that may be evaluated in this article, or claim that may be made by its manufacturer, is not guaranteed or endorsed by the publisher.
